# Causes of Heat in Mines

**Published:** 1881-04

**Authors:** 


### THE CAUSES OF HEAT IN MINES.

Lime is undoubtedly one cause of heat in our mines, but it is not the only nor the great heat producer. Lime is local in its action; the heat produced by it is confined to certain sections of the mines, while underlying the whole length of the Comstock lode is that which causes the general heat, namely, the deposits of iron pyrites. The hottest places in the mines are where the heat is generated by both lime and pyrites; it is the heat from the lime added to the general heat from nature's workshop below.

The hot springs of Colorado may derive a portion of their heat from the decomposition of lime, but this is but a secondary cause. The great and first cause of heat in springs and mines is the decomposition of iron pyrites—masses of iron and sulphur. At Steamboat

Springs and other places in this State, and at most of the hot springs in California, the heat is produced by the burning out or decomposition of iron pyrites. At Steamboat Springs the course of the deposits of iron pyrites is northeast and southwest, the same as that of the great mineral-bearing veins of the State. The line of active springs follows the course of this deposit, moving toward the northeast. At the southwest end are to be seen places where the deposit of iron pyrites and similar minerals carrying large quantities of sulphur has burned out, and the springs have died away. The process of burning out is is slowly moving towards the northeast. In 1860 the writer saw a new spring just starting up through a thick growth of grass in a bit of meadow land far in advance of the older and larger ones, but on the same general line, well out to the northeast.

The base metal deposit at Steamboat Springs has the same dip as the Comstock, and is working east as well as toward the north. By going from half to three-quarters of a mile west of the present active spring at Steamboat, one may see where the springs were ages ago, along near the croppings or upper edge of the deposit or pyritic matter. As the decomposition proceeded downward and eastward along the dip of the deposit, the steam and hot water found or forced new vertical channels of escape. Some of these openings are probably natural crevices, but the majority are undoubtedly rents produced by the force of steam and pent-up gases. Even on the surface of Steamboat Springs are to be seen long rents from an inch or two to over a foot in width that have a northeast and southwest course. In California some of the hot springs are observed to be dying out at one end of their line and advancing into new ground at the other.

At Steamboat Springs we probably see a big mineral vein (like the Comstock) in process of formation. Ages ago there was probably a line of hot springs along the course of the Comstock. The mines of Europe and Mexico, which are comparatively cold at great depths, are undoubtedly ages and ages older than the Comstock. The Comstock is probably the youngest mine in any part of the world that is now known or worked. Here, down in our lower levels, we are following close upon the heels of nature—getting well down into her workshop.

As to the heat-generating power of sulphur and iron those who desire to do so may satisfy themselves. Take a few pounds of iron filing, borings and drillings from a machine shop, wet them and mix in a pound or two of sulphur, then tamp the mixture firmly in a hole into the ground—like a post hole—covering with two or three inches of dirt, and in a short time there will be seen a minature volcano, the batch of iron and sulphur taking fire spontaneously.—*Virginia (Nev.) Enterprise.*

